# Clinical efficacy of five-element music therapy in alleviating psychological distress among patients undergoing maintenance hemodialysis: a multicenter, non-inferiority, randomized controlled trial

**DOI:** 10.3389/fpsyg.2026.1796603

**Published:** 2026-04-13

**Authors:** Bo Zhang, Wen Hao, Luping Cheng, Jiali Zheng, Hui Zeng, Deqiong Xie

**Affiliations:** 1 Department of Clinical Medicine, North Sichuan Medical College, Nanchong, Sichuan, China; 2 Department of Nephrology, Yibin Second People's Hospital, Yibin, Sichuan, China

**Keywords:** five-element music, maintenance hemodialysis, music therapy, psychological distress, relaxation training (RT)

## Abstract

**Background:**

Psychological distress is common in patients undergoing maintenance hemodialysis (MHD) and may compromise quality of life and treatment adherence. Music-based interventions have shown potential benefits for emotional symptoms; however, evidence on Five-Element Music Therapy (FEMT), a Traditional Chinese Medicine-based intervention, remains limited in the MHD population. To our knowledge, no randomized controlled trial (RCT) has directly compared FEMT with relaxation training (RT) in these patients. This study therefore aimed to evaluate the efficacy of FEMT in alleviating psychological distress in patients undergoing MHD and to determine its non-inferiority to RT.

**Methods:**

A parallel-group randomized controlled trial was conducted in 140 patients MHD who met the predefined inclusion and exclusion criteria at hemodialysis centers of three tertiary hospitals in Yibin, China. Participants with psychological distress were randomly assigned in a 1:1 ratio to either the RT group or the FEMT group. Assessments were performed at baseline, posttreatment (week 4), and 1-month follow-up using the validated Distress Thermometer (vDT), Self-Rating Anxiety Scale (SAS), Self-Rating Depression Scale (SDS), Pittsburgh Sleep Quality Index (PSQI), and the Short Form-36 Health Survey (SF-36). The primary outcome was psychological distress measured by the vDT; secondary outcomes included anxiety, depression, sleep quality, and health-related quality of life. Non-inferiority was evaluated on the basis of vDT scores, with a prespecified non-inferiority margin of 1 point.

**Results:**

The trial included 140 participants, including 54 women (38.6%) and 86 men (61.4%), with a mean age of 56.33 ± 12.16 years; 70 participants were assigned to each group. Both groups showed significant reductions in psychological distress over time. The estimated between-group mean difference in vDT score was 0.35 points (90% *CI*, 0.18–0.53) at posttreatment and 1.19 points (90% *CI*, 1.02–1.36) at the 1-month follow-up, with both estimates favoring FEMT. At both time points, the lower bound of the 90% confidence interval (CI) remained above the prespecified non-inferiority boundary, indicating that FEMT met the non-inferiority criterion relative to RT. In addition, because the lower bounds of the 90% confidence intervals were also greater than 0 at both time points, the results were statistically compatible with superiority of FEMT over RT.

**Conclusion:**

Both FEMT and RT were associated with improvements in psychological distress among patients undergoing MHD. FEMT met the prespecified non-inferiority criterion and was statistically compatible with superiority over RT at posttreatment and at the 1-month follow-up. These findings support the short-term clinical value of FEMT as a culturally adapted intervention for psychological distress in patients undergoing MHD.

**Trial registration:**

itmctr.ccebtcm.org.cn, Identifier: ITMCTR2026000143.

## Introduction

Psychological distress, also referred to as emotional distress, is defined by the National Comprehensive Cancer Network (NCCN) as a multifactorial unpleasant emotional experience that may affect psychological (cognitive, behavioral, and emotional), social, and spiritual well-being, ranging from common feelings of vulnerability and sadness to disabling problems such as depression, anxiety, panic, social isolation, and existential crisis (Riba et al., 2019). Epidemiological data indicate that by 2020, approximately 850 million individuals worldwide had chronic kidney disease (CKD), with China accounting for the largest CKD population, estimated at 132.3 million, corresponding to a prevalence of 10.8%. In 2021, nearly 750,000 patients receiving hemodialysis were registered in China. It has been projected that by 2030, the number of patients with end-stage renal disease will exceed 3 million, of whom approximately 1.48 million will receive hemodialysis treatment (Xu et al., 2025). Among patients undergoing maintenance hemodialysis (MHD), the prevalence of psychological distress has been reported to be as high as 52.99%, which may be attributable to the distinctive clinical and psychosocial burden of this population (He, 2022). Patients are typically required to attend hemodialysis centers three times per week, with each session lasting approximately 4 h, thereby substantially disrupting daily life and occupational functioning (Carswell et al., 2021). Moreover, physical discomfort during dialysis, strict dietary and fluid restrictions, uncertainty regarding disease prognosis, loss of social roles, limitations in daily activities, and fear of death may collectively impose a considerable burden on patients. Consequently, mental health (MH) and quality of life are adversely affected, resulting in varying degrees of psychological distress, which may manifest as anxiety, depression, sleep disturbance, fatigue, and somatic symptoms (Hosseini et al., 2024; Legrand et al., 2020). Anxiety and depression are common but frequently underrecognized among patients undergoing MHD (Cohen et al., 2016). Previous studies have shown that 20% to 40% of patients receiving MHD experience depression, with a risk that is three to four times higher than that observed in the general population or in individuals with other chronic diseases (Shirazian et al., 2017). Psychological distress not only adversely affects disease control and clinical outcomes, but also substantially reduces treatment adherence and quality of life. Therefore, the identification of effective interventions to alleviate psychological distress in patients undergoing MHD is of considerable clinical importance.

Relaxation training (RT) induces a state of physical and mental relaxation through structured muscle and cognitive relaxation techniques and has been widely applied in chronic disease settings to alleviate symptoms such as anxiety and tension (Arunraj et al., 2025). In parallel, music therapy has developed rapidly since the mid-20th century, particularly in the United States, where the American Music Therapy Association (AMTA) was established and has become one of the leading professional organizations in this field. At present, music therapy is widely used in a broad range of clinical contexts worldwide. Rooted in the Five Elements theory of Traditional Chinese Medicine (TCM), Five-Element Music Therapy (FEMT) links the five musical tones to the five zang organs and the five emotions, thereby forming a culturally grounded music-based intervention. According to this framework, listening to music in specific modes may harmonize the flow of qi within the internal organs, restore yin–yang balance, regulate emotional states, and ultimately achieve a state in which “yin and yang are harmonized and the spirit is at peace” (Zhang et al., 2022). FEMT is characterized by relative simplicity and clinical feasibility, and it has been reported to improve depression, anxiety, sleep quality, and quality of life in several clinical populations, including patients with cancer, women in the perinatal period, and individuals with post-stroke depression (Yang et al., 2021; Wu et al., 2020; Sun et al., 2024). More recently, randomized studies have suggested that FEMT may also confer benefits in patients MHD (Zhang et al., 2025). More broadly, music-based interventions in hemodialysis settings have been associated with improvements in anxiety, pain, stress, and psychological well-being (Lin et al., 2024). Because patients undergoing MHD experience long-term treatment burden, repeated procedural stress, and substantial symptom burden, FEMT—which is characterized by cultural relevance, favorable acceptability, low implementation burden, and ease of delivery during dialysis sessions—may represent a promising supportive intervention that can be implemented without disrupting routine dialysis care. Against this background, the present study was designed to evaluate the efficacy of FEMT in alleviating psychological distress in patients undergoing MHD and to compare its effects with those of RT.

## Method

### Design

This study was designed as a parallel-group, multicenter, non-inferiority randomized controlled trial (RCT) to evaluate the effect of FEMT on psychological distress in patients undergoing MHD. After completion of the baseline assessment, participants were randomly allocated in a 1:1 ratio to either the FEMT group or the RT group according to a computer-generated randomization sequence. The allocation sequence was generated and managed by an independent statistician, and could not be predicted or altered by the investigators. Changes in psychological distress-related outcomes were compared within and between groups over the course of the study. In addition, all outcome assessors remained blinded to group assignment throughout the trial. The trial was conducted in accordance with the Consolidated Standards of Reporting Trials (CONSORT) guidelines and was approved by the Ethics Committee of Yibin Second People's Hospital (Ethics Approval No. 2023-062-01) (Supplementary material). Written informed consent was obtained from all participants prior to enrollment.

### Participants

All participants were recruited from the hemodialysis centers of Yibin Second People's Hospital, Yibin Third People's Hospital, and Yibin Second Hospital of Traditional Chinese Medicine between August 2023 and August 2025.

The inclusion criteria were as follows: (1) age ≥ 18 years; (2) receipt of maintenance hemodialysis for at least 3 months, with dialysis performed three times per week; (3) adequate ability to read, hear, and understand the questionnaire items, as well as sufficient verbal or written communication skills; (4) a score of ≥5 on the validated Distress Thermometer (vDT); and (5) provision of informed consent by the participant and his or her family members.

The exclusion criteria were as follows: (1) severe cardiac, hepatic, cerebral, or other major systemic diseases; (2) recent surgery; (3) a history of psychiatric disorders; (4) impaired consciousness, cognitive dysfunction, or severe visual or auditory impairment; (5) incomplete clinical records; (6) refusal to cooperate with the investigation or inability to complete the questionnaire; (7) chronic alcohol abuse, substance misuse, or long-term use of opioids or benzodiazepines; and (8) current use of anti-anxiety or antidepressant medications, or receipt of psychotherapy.

### Randomization, blinding

A 1:1 randomization sequence was generated using IBM SPSS Statistics version 26.0. Randomization cards, each containing a serial number, a random number, and a group assignment, were placed into sequentially numbered, sealed, light-proof envelopes. The randomization scheme was managed by research assistants, and neither the participants nor the outcome assessors were aware of the allocation plan before the intervention was initiated. After completion of the baseline assessment, the envelopes were opened in enrollment order, and participants were randomly assigned to either the RT group or the FEMT group. Because of the nature of the interventions, blinding of participants and research assistants was not feasible; therefore, a blinded outcome-assessor and blinded-statistician design was adopted. All scale ratings and efficacy evaluations during the trial were conducted by independent assessors who were not involved in group allocation or intervention delivery. The RT group and FEMT group were coded as Group A and Group B, respectively, and this information remained concealed until completion of the statistical analysis.

### Intervention methods

#### FEMT

The Five-Element Music intervention used in this study was selected from Five Elements Music, published by China Medical Audio-Visual Publishing House and composed and arranged by Shi Feng of the 81st Film Studio, based on the Five Elements theory of TCM. According to the TCM principle of syndrome differentiation and organ-based selection, the Yu-toned (Kidney) suite was used in this study. Within the Five-Element Music system, five therapeutic musical modes are traditionally distinguished: Zhi (Heart), Jiao (Liver), Shang (Lung), Gong (Spleen), and Yu (Kidney). The Yu-toned music is characterized by a relatively soothing and tranquil acoustic profile, which is intended to promote physical and mental relaxation and support emotional regulation.

A standardized playback protocol was adopted using bone-conduction sleep speakers. During each session, patients were instructed to remain in a quiet environment, keep their eyes closed, and maintain a relaxed posture. The playback volume was adjusted to a comfortable and appropriate level. Each session lasted 60 min and was administered three times per week during hemodialysis for four consecutive weeks.

#### RT

(1) Psychological relaxation training: To address pre-existing negative emotional states, including loneliness, anxiety, depression, tension, and fear, the frequency of supportive communication with patients was increased. In addition, audiovisual materials tailored to patients' interests were provided to facilitate relaxation and emotional stabilization. Each session lasted 30 min.
(2) Deep-breathing relaxation training: The patient was placed in the supine position and instructed to allow the whole body to relax into a comfortable state. Gentle abdominal breathing was then performed while the patient was guided to focus attention on the movement of air during inhalation and exhalation. During the exercise, the abdomen was expanded with deep inhalation, the breath was held for 2 s, and exhalation was performed slowly to allow gradual abdominal contraction. An inhalation-to-exhalation ratio of 2:1 was maintained. Each session lasted 30 min and was administered three times per week during hemodialysis for four consecutive weeks.

### Data collection methods and quality control

This study was supervised by an experienced clinical psychologist, who provided standardized training to all evaluators in psychiatric assessment and structured interviewing, and who also reviewed the eligibility of all enrolled participants. General demographic and clinical information were collected prior to the intervention using a self-designed case report form. Participants completed the vDT, Self-Rating Anxiety Scale (SAS), Self-Rating Depression Scale (SDS), Pittsburgh Sleep Quality Index (PSQI), and the Short Form-36 Health Survey (SF-36) at baseline, at the end of the 4-week intervention period, and at the end of the 1-month follow-up. All assessment data were checked on site by the evaluators for completeness and accuracy.

To ensure standardized implementation of the intervention protocol, dedicated research personnel were assigned to both the FEMT and RT groups. Different hemodialysis centers were managed by separate researchers, all of whom received unified training to ensure consistency in intervention delivery. Before each session, for participants in the FEMT group, bone-conduction sleep speakers were distributed, the music was played according to protocol, and the volume was adjusted to an appropriate level. The equipment was collected after each session and redistributed before subsequent sessions. During the intervention, researchers observed whether participants entered the intended relaxation state and provided guidance when adequate relaxation was not achieved. For the RT group, trained instructors supervised the intervention process and provided timely prompts or corrective guidance when necessary to maintain procedural consistency and intervention fidelity.

#### Exclusion, withdrawal, and termination criteria

(1) Voluntary withdrawal at the request of the participant or the participant's family;
(2) Discontinuation of the intervention due to severe adverse events or complications that rendered the participant unable to tolerate treatment;
(3) Poor compliance, defined as completion of fewer than 80% of the scheduled intervention sessions, failure to complete the prespecified baseline or follow-up assessments, and/or other major protocol deviations judged by the investigator to potentially affect the evaluation of treatment efficacy, such as voluntary participation in other similar psychological interventions.

### Outcome measures

#### Baseline characteristics

These included: (1) demographic information, including sex, age, and other basic characteristics; and (2) general clinical information, including dialysis duration, primary kidney disease, number of arteriovenous fistulas, and comorbidities such as hypertension and diabetes.

#### Primary outcome

1) Psychological Distress: The psychological distress screening tool was developed by the NCCN and consists of the Distress Thermometer (DT) and its accompanying Problem List. The DT is scored on an 11-point scale ranging from 0 (no distress) to 10 (extreme distress), with higher scores indicating greater psychological distress (National Comprehensive Cancer Network, 2003). In previous studies, our research team validated the DT for patients with MHD. The results indicated that a score of 5 is the optimal cutoff value for identifying significant psychological distress, making it suitable for rapid screening in this population. Building on this, we further revised the accompanying questionnaire based on the clinical characteristics of MHD patients, resulting in a pool of items tailored for this population, comprising 40 items across 4 dimensions. This study employed the DT, as validated by our research team, to assess the level of psychological distress. The accompanying questionnaire on factors associated with psychological distress was used to identify the sources of patients' psychological distress and was implemented as an auxiliary screening tool; however, it was not included in the analytical framework for the primary or secondary efficacy outcome measures of this study.

#### Secondary outcome

1) Anxiety: Assessed using the SAS, developed by Zung (1971). This 20-item scale primarily evaluates the frequency of anxiety symptoms across various life situations, with higher scores indicating more severe anxiety symptoms.
2) Depression: Assessed using the SDS developed by Zung (1965), which consists of 20 items and primarily evaluates depressive mood, physical symptoms, and psychomotor behaviors across various life situations. Higher scores indicate more severe depressive symptoms.
3) Sleep: Assessed using the PSQI, developed by Buysse et al. (1989). This questionnaire comprises 19 self-rated items and 5 peer-rated items, with 18 items contributing to the score across 7 dimensions. Each dimension is scored from 0 to 3 based on severity, with higher total scores indicating poorer sleep quality.
4) Quality of Life: The SF-36 was used for evaluation. This is a general-purpose health survey developed by the Boston Health Research Institute in the United States, consisting of 36 items. Item 2 is excluded from the score to objectively assess changes in the participants' health over the past year; the remaining 35 items are divided into eight domains: physical function (PF), role physical (RP), Bodily Pain (BP), General Health (GF), Mental Health (MH), Vitality (VT), Social Function (SF), and Role Emotional (RE). These eight dimensions can be broadly categorized into two aspects: physical health (4 dimensions) and mental health (4 dimensions), to assess patients' physical and mental health status. A specific formula is used to convert the scores to a scale of 0–100, with higher scores indicating a higher quality of life (Unalan et al., 2012). Each scale was assessed at three time points: baseline, 4 weeks after treatment, and 1 month follow-up.

Sample Size Calculation


$$\begin{array}{l} \text{N=}\frac{{\text{(Z}\alpha +\text{Z}\beta \text{)}}^{2}\times 2\sigma^{2}}{\delta^{2}} \end{array}$$

Sample size estimation was based on a pilot-study effect size of 0.56. Under the assumptions of a two-sided α = 0.05, β = 0.10, and a 1:1 allocation ratio, the minimum required sample size was calculated to be 68 participants per group (136 in total). After allowing for an anticipated 20% dropout rate, the planned target sample size was increased to 170 participants. However, owing to funding-cycle constraints and slower-than-expected recruitment, only 140 participants were ultimately randomized. Because only four participants withdrew after randomization, the final analyzable sample remained above the minimum sample size required under the pilot-based assumptions.

### Statistical analysis

Statistical analyses were performed using IBM SPSS Statistics version 26.0, and figures were generated using GraphPad Prism version 10.6.0. The Shapiro–Wilk test was used to assess the normality of continuous variables. Variables with a normal distribution were presented as mean ± standard deviation (SD), whereas non-normally distributed variables were expressed as median (interquartile range, IQR). Between-group comparisons were conducted using the independent-samples *t*-test or the appropriate non-parametric test, as applicable. Categorical variables were summarized as frequencies and percentages. All efficacy analyses were conducted according to the intention-to-Treat principle (ITT). Repeated-measures mixed-effects regression models were fitted using maximum likelihood estimation under the assumption that data were missing at random. For the primary outcome, the model included fixed effects for group, time, and the group-by-time interaction, together with a random intercept for each participant. The group-by-time interaction term was used to estimate between-group differences at each post-baseline time point, including the 1-month follow-up. For the primary outcome, the between-group difference in vDT score was defined as RT-FEMT. Because a lower vDT score indicates less psychological distress, a positive value indicates a result favorable to FEMT. With a prespecified non-inferiority margin of 1 point, the corresponding statistical non-inferiority boundary under this coding scheme was −1 point. Accordingly, when the lower limit of the two-sided Wald 90% confidence interval (CI) exceeded −1, FEMT was considered non-inferior to RT; when the lower limit further exceeded 0, the result was considered statistically compatible with superiority of FEMT over RT. The non-inferiority margin was determined on the basis of clinical judgment and the *a priori* sample size assumptions. For each continuous secondary outcome, a linear mixed-effects model was fitted with fixed effects for time, treatment group, and the group-by-time interaction, together with a random intercept for each participant. Residual diagnostics were performed on the models to assess whether the assumptions of linear regression were met. Residuals were extracted from each model, and normality was assessed using the Shapiro–Wilk test and Q–Q plots; homoscedasticity was tested using scatter plots of residuals against fitted values. The results showed that the residuals approximately followed a normal distribution (*P* > 0.05 for the Shapiro–Wilk test in all models), the Q–Q plots did not show any significant deviation from the diagonal line, and no obvious violations of the model assumptions were observed. Therefore, no variable transformations were performed, and the models can be considered to satisfy the normality assumption.

## Results

### Participants

**Figure 1:**
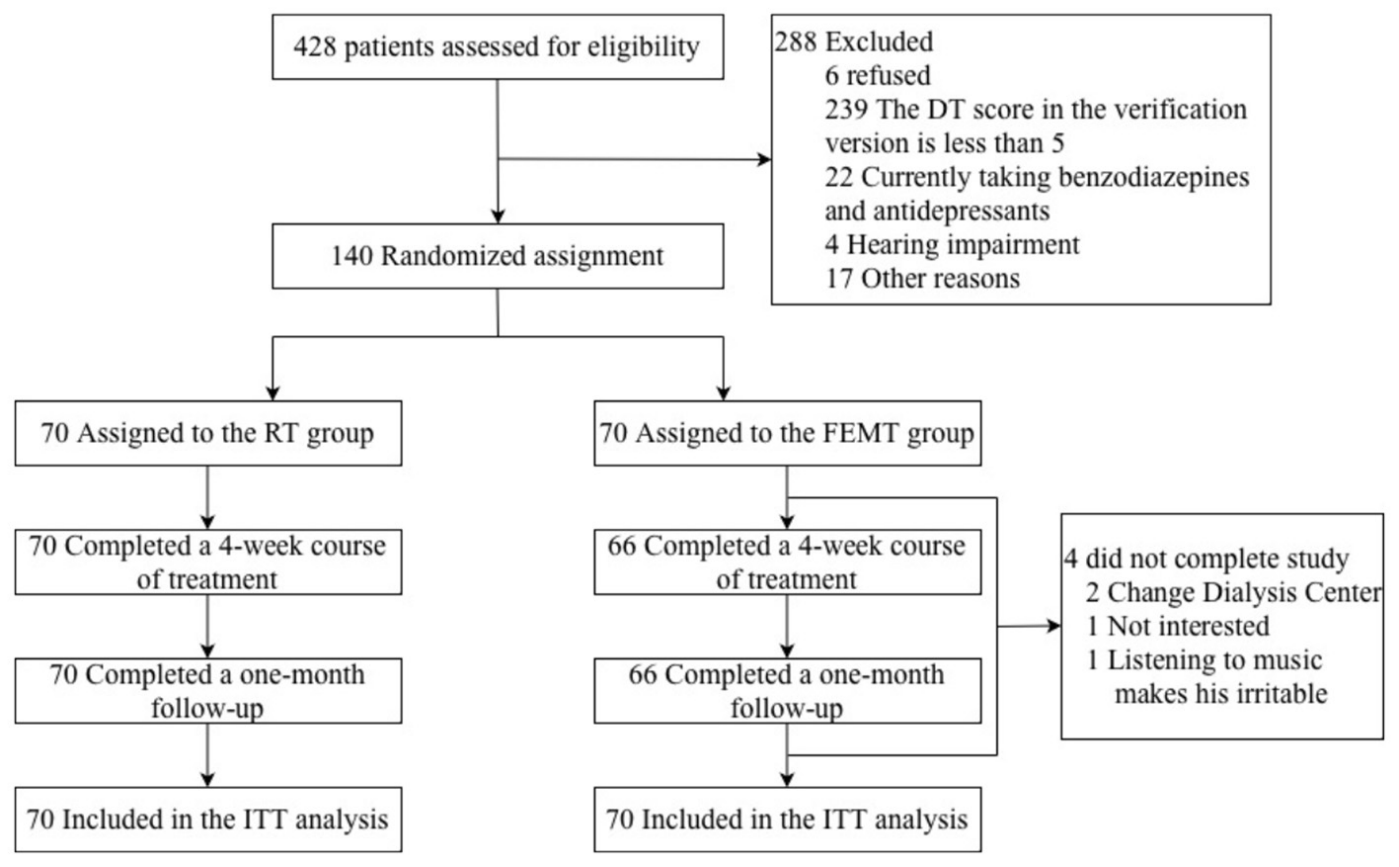
Participant flow through the trial. FEMT, five-element music therapy; RT, relaxation training.

Of the 428 adults screened for eligibility, 288 were excluded, and 140 participants were ultimately enrolled in the trial between August 2023 and August 2025 (Yibin Second People's Hospital Hemodialysis Center, *n* = 87; Yibin Third People's Hospital Hemodialysis Center, *n* = 30; Yibin Second Hospital of Traditional Chinese Medicine, *n* = 23; Figure 1). Seventy participants were randomly allocated to the FEMT group and 70 to the RT group. Participants' sociodemographic and clinical characteristics are summarized in Table 1. Baseline comparisons showed no statistically significant between-group differences in vDT, SAS, SDS, PSQI, or SF-36 scores before the intervention (all *P* > 0.05; Table 2).

**Table 1:** Participant characteristics at baseline.

Characteristic	*T* (*n* = 140)	RT group (*n* = 70)	FEMT group (*n* = 70)
Age	56.33 (12.16)	56.93 (12.69)	55.73 (11.67)
Sex			
Male	86 (61.4%)	43 (61.4%)	43 (61.4%)
Female	54 (38.6%)	27 (38.6%)	27 (38.6%)
Education level			
Junior high school and below	86 (61.4%)	41 (58.6%)	45 (64.3%)
High school and above	54 (38.6%)	29 (41.4%)	25 (35.7%)
Medical payment methods			
Resident medical insurance	89 (63.6%)	43 (61.4%)	46 (65.7%)
Employee medical insurance	51 (36.4%)	27 (38.6%)	24 (34.3%)
Primary disease			
Glomerulonephritis	66 (47.1%)	39 (55.7%)	27 (38.6%)
Diabetic nephropathy	21 (15%)	8 (11.4%)	13 (18.6%)
Hypertensive nephropathy	6 (4.3%)	2 (2.9%)	4 (5.7%)
IgA nephropathy	3 (2.1%)	1 (1.4%)	2 (2.9%)
Polycystic kidney disease	10 (7.1%)	3 (4.3%)	7 (10%)
Systemic lupus erythematosus	2 (1.4%)	2 (2.9%)	0 (0)
Unclear	32 (22.9%)	15 (21.4%)	17 (24.3%)
Complication^a^			
0	27 (19.3%)	16 (22.9%)	11 (15.7%)
1	82 (58.6%)	41 (58.6%)	41 (58.6%)
2	31 (22.1%)	13 (18.6%)	18 (25.7%)
Dialysis duration			
4 months−1 years	18 (12.9%)	6 (8.6%)	12 (17.1%)
1–5 years^b^	76 (54.3%)	37 (52.9%)	39 (55.7%)
5–10 years^c^	32 (22.8%)	19 (27.1%)	13 (18.6%)
>10 years	14 (10%)	8 (11.4%)	6 (8.6%)
Number of fistulas	1.47 (0.79)	1.6 (0.86)	1.34 (0.70)

^a^Complications include two types: hypertension and diabetes.

^b^Includes 1 year but not 5 years.

^c^Includes 5 years but not 10 years.

**Table 2:** Comparison of scores on each scale between the two baseline groups.

Scale	RT group (*n* = 70)	FEMT group (*n* = 70)	*Z*	*P*
DT score, points	6.00 (5.00, 7.00)	6.00 (5.75, 8.00)	−1.012^a^	0.311
SAS score, points	43.75 (36.25, 53.75)	44.38 (36.25, 56.25)	−0.601^a^	0.548
SDS score, points	55.00 (47.50, 65.31)	61.25 (50.00, 66.25)	−1.004^a^	0.315
PSQI score, points	13.00 (6.75, 14.00)	14.00 (7.75, 16.00)	−1.951^a^	0.051
SF-36 score, points				
PF	70.00 (60.00, 75.00)	75.00 (65.00, 85.00)	−1.830^a^	0.067
SF	22.22 (22.22, 58.33)	22.22 (22.22, 44.44)	−0.929^a^	0.353
RP	0.00 (0.00, 25.00)	0.00 (0.00, 25.00)	−0.608^a^	0.543
BP	74.00 (41.00, 100.00)	74.00 (41.00, 90.63)	−0.694^a^	0.488
VT	50.00 (35.00, 65.00)	45.00 (35.00, 60.00)	−0.986^a^	0.324
RE	0.00 (0.00, 75.00)	0.00 (0.00, 66.67)	−0.389^a^	0.165
MH	60.00 (48.00, 68.00)	60.00 (48.00, 68.00)	−1.276^a^	0.202
GH	32.50 (20.00, 45.00)	33.50 (20.00, 45.00)	−0.248^a^	0.804

^a^Mann–Whitney *U*-test.

### Primary outcome

**Figure 2:**
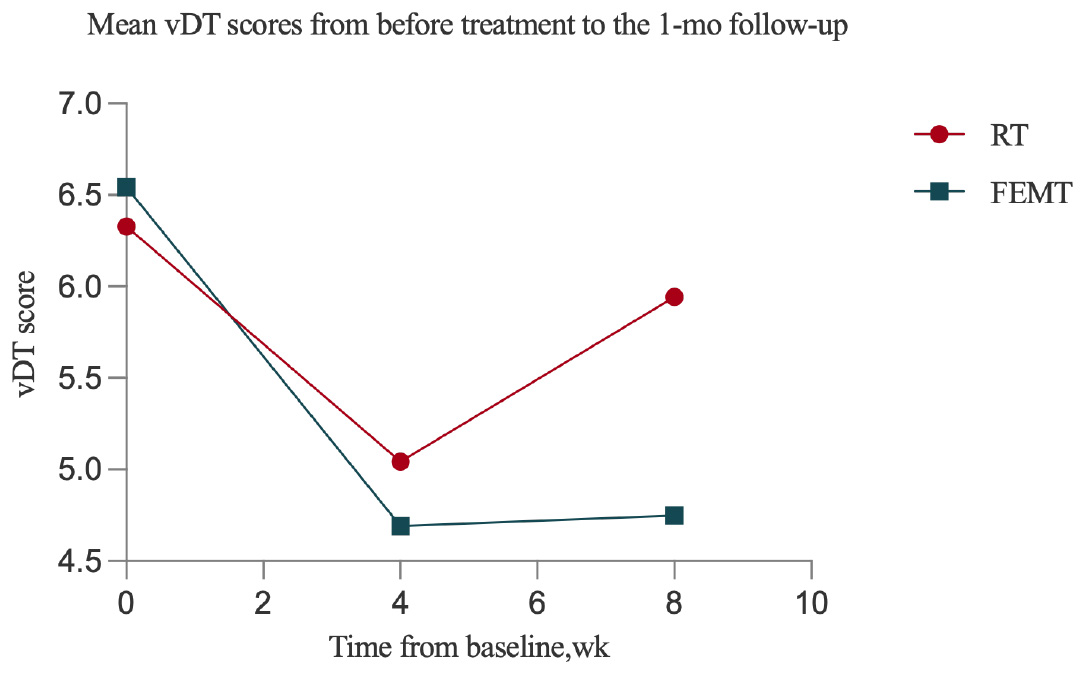
Trends in estimated mean changes in validated psychological distress thermometer scores for the two groups at baseline, at the end of the intervention, and at follow-up.

**Figure 3:**
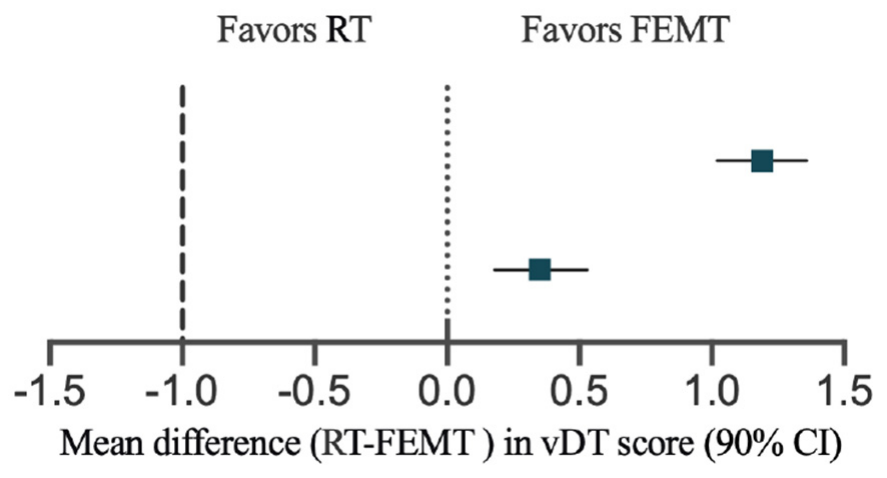
Estimated between-group differences in vDT scores (RT – FEMT) and their 90% confidence intervals at the end of the intervention and at the 1-month follow-up. Since a lower vDT score indicates less psychological distress, positive values indicate a benefit for FEMT. Solid vertical lines indicate the line of no difference between groups (0), and dashed vertical lines indicate the pre-specified non-inferiority margin (−1 point).

Figure 2 and Table 3 present the changes in vDT scores in the FEMT and RT groups from baseline to the end of the intervention and to the 1-month follow-up. The results showed that vDT scores in both groups declined significantly from baseline to the end of the intervention and remained lower than baseline at the 1-month follow-up, with large within-group effect sizes. At the end of the intervention, the estimated mean difference in vDT scores was 0.35 points (90% *CI*, 0.18–0.53), indicating that the FEMT group had lower levels of psychological distress than the RT group. At the 1-month follow-up, the estimated mean difference was 1.19 points (90% *CI*, 1.02–1.36), and the result at this time point also favored FEMT. Because the lower bounds of the 90% confidence intervals at both time points were greater than −1, FEMT met the prespecified non-inferiority criterion relative to RT. Furthermore, because the lower bounds of the 90% confidence intervals at both time points were greater than 0, the findings at posttreatment and at the 1-month follow-up were statistically compatible with superiority of FEMT over RT under the prespecified analytical framework. Nevertheless, the 1-month follow-up still represents short-term observation. Accordingly, although these findings suggest that the relative advantage of FEMT persisted for at least 1 month, they do not permit inference regarding longer-term durability of effect. The corresponding between-group comparisons are shown in Figure 3 and Table 4. In addition, the results of the residual diagnostics indicate that the assumptions of normality and homoscedasticity in the mixed-effects model are generally satisfied, and no significant deviations from the model assumptions were observed.

**Table 3:** Within-group estimated means and effect sizes vDT.

Time	Change from pretreatment^a^, *B* (*SE*)		Within-group effect size^b^. Cohen's *d* [95% *CI*]	
	RT group	FEMT group	RT group	FEMT group
Post	−1.29 (0.10)	−1.85 (0.11)	−2.04 [−2.45, −1.63]	−2.94 [−3.42, −2.46]
1-month follow-up	−0.39 (0.10)	−1.79 (0.11)	−0.61 [−0.95, −0.27]	−2.85 [−3.32, −2.38]

^a^Using a DT mixed model with fixed effects for time, group, and their interaction, along with a random intercept, compare the estimated mean differences within each group to pre-treatment levels.

^b^Calculate the effect size within groups using the estimated means from the same mixed-effects model, and use the residual standard deviation from the random effects as sigma.

**Table 4:** The estimated between-group mean difference in vDT between the two groups at the time of intervention and at the 1-month follow-up.

Time	Mean difference	90% *CI*
Posttreatment	0.35	[0.18, 0.53]
1-month follow-up	1.19	[1.02, 1.36]

### Secondary outcomes

Based on the mixed-effects models, the patterns of between-group differences in the secondary outcomes were not fully consistent across time points.

Regarding anxiety symptoms, at the end of the intervention, the adjusted mean SAS score in the FEMT group was significantly lower than that in the RT group (Cohen's *d* = −0.68, 95% *CI*: −1.02– −0.34), indicating that the result at this time point favored FEMT. However, at the 1-month follow-up, the direction of the between-group difference reversed, with the adjusted mean SAS score in the FEMT group being significantly higher than that in the RT group (Cohen's *d* = 0.45, 95% *CI*: 0.11–0.78), indicating that the between-group difference observed at the end of the intervention was not sustained at follow-up.

With regard to depressive symptoms, no significant between-group difference in SDS scores was observed at the end of the intervention (Cohen's *d* = 0.05, 95% *CI*: −0.28–0.39). However, at the 1-month follow-up, the adjusted mean SDS score in the FEMT group was significantly lower than that in the RT group (Cohen's *d* = −1.58, 95% *CI*: −1.96–1.20), indicating that the result at this follow-up time point favored FEMT.

With regard to sleep, at the end of the intervention, the adjusted mean PSQI score in the FEMT group was significantly lower than that in the RT group (Cohen's *d* = −0.36, 95% *CI*: −0.70–0.03), indicating a result favorable to FEMT at that time point. However, at the 1-month follow-up, the adjusted mean PSQI score in the FEMT group was significantly higher than that in the RT group (Cohen's *d* = 1.36, 95% *CI*: 0.99–1.73), suggesting that the between-group difference observed at the end of the intervention was not maintained at follow-up.

Regarding quality of life, the results across the SF-36 dimensions showed a degree of heterogeneity. No significant between-group differences were observed for PF and RP at the end of the intervention; however, at the 1-month follow-up, the adjusted mean scores in the FEMT group were significantly higher than those in the RT group for both dimensions. The between-group differences observed for GH and SF at the end of the intervention were no longer significant at follow-up. VT, RE, and MH showed between-group differences favoring FEMT at the end of the intervention, and these differences remained evident at the 1-month follow-up. No significant between-group differences were observed for BP throughout the study period.

Overall, the effects of FEMT on psychological symptoms and quality-of-life outcomes appeared to vary by both time point and dimension. For some measures, between-group differences were observed only at the end of the intervention and did not persist at the 1-month follow-up; for others, these differences emerged only at follow-up or remained detectable during the short-term follow-up period (Table 5).

**Table 5:** Intra-group changes in secondary outcome measures at each follow-up time point and standardized mean differences between groups.

Outcome	Change from pretreatment, *B* (*SE*)^a^		Group contrasts, Cohen *d* (95% *CI*)^b^
	RT group	FEMT group	FEMT group vs. RT group
SAS			
Posttreatment	−2.75 (0.28)	−5.06 (0.28)	−0.68 [−1.02, −0.34]
1-month follow-up	−1.38 (0.28)	−1.84 (0.28)	0.45 [0.11, 0.78]
SDS			
Posttreatment	−5.73 (0.37)	−7.56 (0.38)	0.05 [−0.28, 0.39]
1-month follow-up	−0.23 (0.37)	−5.67 (0.38)	−1.58 [−1.96, −1.20]
PSQI			
Posttreatment	−0.04 (0.40)	−2.42 (0.41)	−0.36 [−0.70, −0.03]
1-month follow-up	0.07 (0.40)	1.80 (0.41)	1.36 [0.99, 1.73]
SF-36			
PF			
Posttreatment	5.43 (1.82)	0.34 (1.84)	0.07 [−0.27, 0.40]
1-month follow-up	0.79 (1.84)	1.51 (1.84)	0.64 [0.30, 0.98]
RP			
Posttreatment	−2.14 (2.75)	3.10 (2.79)	0.28 [−0.05, 0.61]
1-month follow-up	−1.43 (2.75)	5.71 (2.79)	0.40 [0.06, 0.73]
BP			
Posttreatment	−1.01 (2.17)	3.57 (2.19)	0.12 [−0.21, 0.45]
1-month follow-up	0.00 (2.17)	3.07 (2.17)	0.00 [−0.33, 0.33]
GH			
Posttreatment	−2.43 (1.64)	4.89 (1.65)	0.76 [0.42, 1.09]
1-month follow-up	1.86 (1.64)	2.00 (1.66)	0.01 [−0.32, 0.34]
VT			
Posttreatment	6.29 (1.76)	15.91 (1.78)	0.92 [0.57, 1.27]
1-month follow-up	1.86 (1.76)	15.01 (1.79)	1.26 [0.90, 1.62]
SF			
Posttreatment	−1.27 (1.93)	4.25 (1.95)	0.48 [0.15, 0.82]
1-month follow-up	3.33 (1.93)	1.71 (1.96)	−0.14 [−0.48, 0.19]
RE			
Posttreatment	24.76 (4.53)	49.58 (4.58)	0.57 [0.23, 0.91]
1-month follow-up	5.71 (4.53)	46.15 (4.58)	1.15 [0.80, 1.51]
MH			
Posttreatment	2.91 (1.36)	10.63 (1.38)	0.63 [0.29, 0.97]
1-month follow-up	1.08 (1.36)	10.57 (1.38)	0.85 [0.51, 1.20]

^a^Baseline-to-follow-up changes were estimated using least squares means from a mixed-effects model with random intercepts, fixed effects for time and group, and an interaction effect between time and group. B was estimated using comparisons of post-treatment minus baseline within each group and follow-up minus baseline.

^b^The effect size between groups was calculated using the least squares mean from the mixed-effects model and the standard deviation of the random effects residuals as sigma. This comparison reflects the standardized difference between the FEMT group and the RT group at the same assessment time point. Comparisons are presented as the FEMT group minus the RT group. For SAS, SDS, and PSQI scores, a decrease in scores indicates improvement; therefore, a negative value indicates that the adjusted mean for the FEMT group at that time point is lower than that of the RT group. For the SF-36 dimensions, an increase in total scores indicates improvement; therefore, a positive value indicates that the adjusted mean for the FEMT group at that time point is higher than that of the RT group.

## Discussion

This RCT is the first to systematically compare the effects of FEMT and RT on psychological distress in patients undergoing MHD. Overall, psychological distress improved in both groups following the intervention, and this improvement remained evident at the 1-month follow-up. Compared with RT, FEMT showed a more favorable overall pattern in the primary outcomes; this advantage was observed immediately after the intervention and persisted through the 1-month follow-up. Regarding the secondary outcomes, FEMT demonstrated more favorable patterns of change in anxiety, depression, sleep, and quality of life at certain time points and across specific dimensions; however, these effects were not uniform, and some heterogeneity was observed across measures and time points. These findings are generally consistent with the overall trends suggested by previous systematic reviews and meta-analyses of music interventions in hemodialysis settings (Lin et al., 2024).

In terms of intervention characteristics, FEMT primarily relies on participants listening attentively and maintaining a relaxed state in a relatively quiet environment, whereas RT typically requires participants to perform active exercises such as guided breathing, muscle relaxation, or meditation. Accordingly, RT may place higher demands on participants' active engagement, proficiency in the exercises, and adherence, which may partly explain the relative advantage of FEMT in dialysis settings. At the same time, because the outcome measures were primarily based on self-report instruments, expectation effects and response bias cannot be completely excluded. This issue may be particularly relevant for culturally meaningful interventions such as FEMT, for which participants' pre-existing beliefs, preferences, and familiarity may have influenced their subjective perceptions of benefit. From a physiological perspective, alterations in mitochondrial function and metabolism may be involved in emotion regulation (Gebara et al., 2021), whereas the ultra-low-frequency acoustic characteristics of FEMT have also been hypothesized to contribute to cell growth and enhanced mitochondrial energy production (Feng et al., 2022). Taken together, these prior findings provide a possible theoretical framework suggesting that FEMT may influence emotion-related symptoms through physiological regulatory pathways. However, because no physiological biomarkers were measured in the present study, it remains unclear whether mitochondrial function, metabolic regulation, or other relevant biological pathways mediated the symptom improvements observed with FEMT. Accordingly, these mechanistic interpretations should be regarded as theoretical and exploratory rather than as direct evidence explaining the clinical effects observed in this trial. Future studies should incorporate biomarkers, neuroendocrine indicators, or other objective physiological measures to more directly examine these potential mechanisms. Overall, as a non-pharmacological intervention with strong cultural adaptability and a low implementation burden, FEMT may have potential clinical value in alleviating psychological distress and related symptoms in patients undergoing MHD. Nevertheless, the consistency of its benefits and the underlying mechanisms require further confirmation in studies with longer follow-up and more rigorous methodological control.

However, a systematic review and meta-analysis of 22 randomized controlled trials reported that FEMT significantly alleviated depressive symptoms in patients with cancer, but did not show a significant effect on anxiety symptoms (Yang et al., 2021). This finding differs from the short-term reduction in anxiety symptoms observed with Five-Element music in the present study. Several factors may account for this discrepancy. First, the present study adopted a more standardized intervention protocol, with all interventions administered during dialysis sessions under the direct supervision and quality control of trained personnel. Second, in accordance with the TCM principle of syndrome differentiation, the musical mode selected in this study was tailored to the characteristics of kidney disease, thereby reflecting a more individualized intervention approach. In contrast, many previous studies did not adequately standardize the selection of musical modes within the Five-Element system. With regard to sleep, the present study found that FEMT was associated with improved sleep quality in the short term; however, this effect was not maintained at follow-up. This finding may be related to the relatively short intervention duration, as the 4-week treatment period may have been insufficient to achieve a stable effect. Previous studies have suggested that regular music-based interventions lasting longer than 4 weeks may be associated with more stable improvements in sleep quality (Chen et al., 2021). Future studies should further examine the mechanisms through which Five-Element music may influence sleep and clarify whether this approach has complementary or distinct advantages relative to other sleep-focused interventions. Such work may provide a more comprehensive basis for understanding the potential clinical applications of music-based interventions in sleep management. A systematic review and meta-analysis also found that music therapy did not substantially improve patients' physical condition or fatigue (Gao et al., 2019), which is consistent with the findings of the present study. Across different dimensions of quality of life, FEMT was associated with varying degrees of improvement. Specifically, no statistically significant between-group differences were observed for PF (*P* = 0.855), RP (*P* = 0.267), or BP (*P* = 0.104). This may indicate that FEMT has limited effects on certain symptom domains. One possible explanation is the complexity of symptom burden in patients with end-stage disease, particularly those undergoing MHD. These patients often present with multiple comorbidities, which may reduce responsiveness to a single therapeutic approach (Yousif et al., 2023; Ai et al., 2022). Moreover, symptom-related improvement is often gradual and may require longer and more sustained intervention to become detectable.

In summary, in the treatment and care of patients undergoing MHD, attention should be directed not only toward survival outcomes but also toward psychological well-being. FEMT showed favorable effects on anxiety, depression, sleep quality, and several domains of quality of life in this study. Given its relative ease of implementation, safety, low cost, and high acceptability, FEMT may represent a clinically promising supportive intervention. Future studies should include larger sample sizes, longer intervention periods, and exploration of potential synergistic effects between FEMT and other approaches, such as psychological interventions and physical rehabilitation. Such efforts may facilitate the development of more integrated intervention strategies to address both psychological and physical symptom burden in this population.

### Advantages and limitations

The trial was characterized by high participant retention and minimal missing data. Both interventions were implemented strictly in accordance with the study protocol, with good adherence, and yielded effect sizes comparable to those reported in previous studies of similar interventions. In addition, this study selected specific Five-Element music pieces according to Zang–Fu syndrome differentiation, thereby reflecting an individualized modal-matching strategy and providing preliminary insights into the standardized and personalized implementation of FEMT. Furthermore, the multicenter design enhanced the representativeness and reliability of the data, thereby allowing a more comprehensive evaluation of the efficacy of FEMT.

Nevertheless, several limitations should be acknowledged. Because both the primary and secondary outcomes were based on self-report instruments, the possibility of expectation effects and response bias cannot be fully excluded. This issue may be particularly relevant for culturally meaningful interventions such as FEMT, for which participants' pre-existing beliefs, preferences, and familiarity may influence their subjective perceptions of benefit. Although outcome assessors were blinded, participants could not be fully blinded to the intervention received, which may have further increased the likelihood that subjective outcome assessments were influenced by non-specific factors. In addition, the follow-up period was limited to 1 month; therefore, evidence regarding the durability of the observed effects remains preliminary and does not permit definitive conclusions regarding longer-term maintenance. Finally, although the study was conducted across multiple centers, all participants were recruited from centers in China, which may limit the generalizability of the findings to other cultural contexts and healthcare settings.

## Conclusion

FEMT was associated with more favorable effects on psychological distress in patients undergoing MHD, underscoring the potential clinical value of culturally adapted interventions. Future studies should further examine its longer-term effects and underlying mechanisms in order to optimize its integration into hemodialysis care.

## Data Availability

The original contributions presented in the study are included in the article/Supplementary material, further inquiries can be directed to the corresponding author.
